# Differences in Secure Messaging, Self-management, and Glycemic Control Between Rural and Urban Patients: Secondary Data Analysis

**DOI:** 10.2196/32320

**Published:** 2021-11-19

**Authors:** Stephanie A Robinson, Dane Netherton, Mark Zocchi, Carolyn Purington, Arlene S Ash, Stephanie L Shimada

**Affiliations:** 1 Center for Healthcare Organization and Implementation Research VA Bedford Healthcare System Bedford, MA United States; 2 The Pulmonary Center Boston University School of Medicine Boston, MA United States; 3 The Heller School for Social Policy and Management Brandeis University Waltham, MA United States; 4 Division of Health Informatics and Implementation Science Population and Quantitative Health Sciences University of Massachusetts Medical School Worcester, MA United States; 5 Department of Health Law, Policy, and Management Boston University School of Public Health Boston, MA United States

**Keywords:** diabetes, secure messaging, rural, self-management, patient portal, urban, data, access, risk, portal, eHealth, digital health, messaging, support, accessible, cross-sectional, veteran

## Abstract

**Background:**

Rural patients with diabetes have difficulty accessing care and are at higher risk for poor diabetes management. Sustained use of patient portal features such as secure messaging (SM) can provide accessible support for diabetes self-management.

**Objective:**

This study explored whether rural patients’ self-management and glycemic control was associated with the use of SM.

**Methods:**

This secondary, cross-sectional, mixed methods analysis of 448 veterans with diabetes used stratified random sampling to recruit a diverse sample from the United States (rural vs urban and good vs poor glycemic control). Administrative, clinical, survey, and interview data were used to determine patients’ rurality, use of SM, diabetes self-management behaviors, and glycemic control. Moderated mediation analyses assessed these relationships.

**Results:**

The sample was 51% (n=229) rural and 49% (n=219) urban. Mean participant age was 66.4 years (SD 7.7 years). More frequent SM use was associated with better diabetes self-management (*P*=.007), which was associated with better glycemic control (*P*<.001). Among rural patients, SM use was indirectly associated with better glycemic control through improved diabetes self-management (95% CI 0.004-0.927). These effects were not observed among urban veterans with diabetes (95% CI –1.039 to 0.056). Rural patients were significantly more likely than urban patients to have diabetes-related content in their secure messages (*P*=.01).

**Conclusions:**

More frequent SM use is associated with engaging in diabetes self-management, which, in turn, is associated with better diabetes control. Among rural patients with diabetes, SM use is indirectly associated with better diabetes control. Frequent patient-team communication through SM about diabetes-related content may help rural patients with diabetes self-management, resulting in better glycemic control.

## Introduction

### Background

Over 30 million people in the United States have been diagnosed with type 2 diabetes [1]. Poor glycemic control, defined as hemoglobin A_1c_ (HbA_1c_) > 8% (64 mmol/mol) [2], in patients with type 2 diabetes is a risk factor for the development of diabetes-related complications including retinopathy, neuropathy, heart disease, stroke, blindness, kidney failure, and lower limb amputations [3]. Costs for diabetes care are high and rising [4,5]. Within the United States, total costs have been estimated at US $465.2 billion, including morbidity, mortality, and medical costs [6]. Glycemic control is the primary therapeutic objective for the prevention of diabetes-related complications [7].

### Diabetes Management in Rural Populations

Diabetes is a nationwide epidemic, though difficulty managing this complex, chronic condition varies across the United States [8]. Management is markedly more difficult in rural communities with limited access to health information and specialty care [9,10]. Diabetes is nearly 10% more prevalent in rural than in urban areas, likely owing to greater risk factors including lower income, older age, and higher body mass index [11]. In addition, individuals living with diabetes in rural areas face numerous barriers (limited availability of diabetes education [12], reduced cell phone coverage and internet access [13], transportation barriers, and lengthy travel distances [14,15]), preventing patients from accessing health care [16]. The Veterans’ Affairs (VA) Office of Rural Health estimates nearly 5 million veterans live in rural areas where access to care can be difficult [17], and that almost 40% of Veterans Health Administration (VHA) patients with diabetes live in rural areas [18].

### Promise of Patient Portals

Diabetes self-management behaviors (eg, medication adherence, diet, physical activity, and monitoring blood glucose levels [19]) are consistently linked to achieving glycemic control. Accessible communication, via face-to-face visits or technology, with providers is essential to foster patients’ disease self-management [20]. Access to diabetes self-management education and ongoing support can be improved by using digital health solutions [21]. Previous research highlights the benefits of using web-based patient portals, suggesting that increased access to information and support may engage patients in the management of their disease and improve health outcomes [22,23]. Considering the access challenges rural patients face, virtual care services may be even more critical in this population for effective diabetes self-management. Features such as secure messaging (SM) in the VHA web-based patient portal My Health*e*Vet (MHV), are fundamental to the goal of increasing access to care. SM can be used in lieu of telephone or in-person visits, or to provide additional opportunities for patient-provider communication between visits. Previous coding of SM content revealed wide variety in how it is used, including self-management behaviors such as medication renewal/refill requests, scheduling, referrals, and discussing medication or health issues [24,25].

Research to date suggests that SM use is associated with higher odds of meeting HbA_1c_ control targets, with increased odds of control for every additional message sent per year [26], and with more years of use [27]. SM use may support improved diabetes self-management, though the exact mechanism among these 3 constructs has not been established. It is also unclear to what extent patient characteristics, such as where they live, may play a role in the effectiveness of SM. SM is potentially more beneficial for rural patients with reduced access to in-person care, though it is also possible that it may be less helpful or accessible for those in rural areas with more limited internet access [28-30].

### This Study

This study examined and compared the benefits of sustained SM use for rural and urban patients with diabetes. Rural patients with diabetes are less likely to engage in self-management behaviors, have worse glycemic control, and more limited access to health care. Therefore, they may depend more on accessible communication to help manage their disease. This study uses a framework that was initially developed to evaluate how the BlueButton within the MHV patient portal can support key stakeholder (eg, patients’) experiences, processes of care (eg, patient-team communication, self-management, and care coordination), and health outcomes, and understanding how contextual characteristics (eg, environment or setting in which patients seek and receive health care) shape use of the technology [31]. We have adapted this framework to evaluate other MHV features including SM.

This study had 3 objectives. We sought to investigate whether diabetes self-management mediates the relationship between SM use and glycemic control (objective 1). Additionally, we sought to understand if this mediation was conditional on the patient’s environment (eg, where the patient lived; objective 2). Finally, we wanted to understand how patients are using SM for diabetes management (objective 3).

## Methods

### Study Design and Recruitment

This retrospective observational, cohort, sequential, explanatory, mixed methods (QUAN qual) study included US veterans living with type 2 diabetes. Table 1 specifies the timeline and sources of sampling and data collection. All participants experienced uncontrolled diabetes in 2012 (defined as mean HbA_1c_>8.0% and less than 25% of the year with an HbA_1c_<8.0%). All participants were sustained users of MHV between 2013 and 2017, defined as having used the portal repeatedly (used prescription refills, viewed or downloaded their health information, and used SM at least twice a year for 2 years between 2013 and 2015) and recently (sent at least 4 SMs between January 2016 and June 2017). Seeking a diverse sample of users who were either in good or poor control of their HbA_1c_, we randomly selected a sample of 500 patients who had achieved good HbA_1c_ control in 2016 (defined as mean HbA_1c_<8.0% for 75% of the year or more) and 500 who remained in poor HbA_1c_ control in 2016 (defined as mean HbA_1c_>8.0% for 75% of the year or more). We mailed the randomly selected participants (N=1000) surveys in November 2017, and an additional 200 surveys at the beginning of 2018.

Quantitative methods were used to examine the associations among SM use in 2017, diabetes self-management between November 2017 and February 2018, mean glycemic control in 2018, and differences between rural and urban patients. Data on patients’ use of the MHV patient portal, their glycemic control, in-person health care utilization, and demographic variables were obtained from the VHA Corporate Data Warehouse (CDW) and merged with survey responses.

Qualitative methods were used to further understand how participants were using SM for diabetes self-management. Purposeful sampling was used to identify 40 survey respondents to participate in semistructured interviews about their diabetes management and technology use. In the survey, participants were asked an open-ended survey question, “Can you tell us about an ‘A-Ha!’ Moment when you realized you could use the MHV portal to better manage your diabetes?” We selected interviewees to represent a variety of responses to this and other survey items about MHV use, including those who used a variety of MHV portal features, those with controlled and uncontrolled diabetes, urban and rural patients, and those with or without comorbid mental health diagnoses. Women and minority veterans were oversampled to broaden the representation of patient demographics. More details regarding our survey sampling methodology [25] and qualitative sampling methodology [32] are available elsewhere. This study was approved by the local institutional review board.

**Table 1:** Study timeline and data sources.

Year	Sampling: diabetes control	Sampling: portal use	Mixed methods data sources	Constructs (source)	Covariates (source)
2012	100% Uncontrolled diabetes	Repeated portal use^a^	—^b^	—	—
2013	—	Repeated portal use^a^	—	—	—
2014	—	Repeated portal use^a^	—	—	—
2015	—	Repeated portal use^a^	—	—	—
2016	50% Achieved control/50% remained uncontrolled	Current portal use^c^	—	—	—
2017	—	Current portal use^c^	Quantitative: survey^d^ Quantitative: corporate Data Warehouse (CDW)	Rurality (CDW) SM use (CDW) Diabetes self-management (Survey)	In-person health care Utilization (CDW) Income (survey) Race (survey)
2018	—	—	Qualitative: semistructured interviews	Hemoglobin A_1c_% time in control (CDW)	—

^a^Defined as having used prescription refills, having viewed or downloaded their health information, and having used secure messaging at least twice a year for 2 years between 2013 and 2015.

^b^—: Not available.

^c^Defined as having sent at least 4 secure messages between January 2016 and June 2017.

^d^Disseminated at the end of 2017 or in early 2018.

### Measures

#### Rurality

We identified rurality on the basis of zip codes recorded in the patient’s address data from the CDW. The VA uses the Rural-Urban Commuting Areas (RUCA) system to define patient residence as either urban (at least 30% of the population residing in an urbanized area as defined by the Census Bureau), highly rural (less than 10% commutes to any community larger than an urbanized cluster), or rural (land areas not defined as urban or highly rural). RUCA codes are created using a validated algorithm developed by the US Department of Agriculture–Economic Research Service to classify US census tracts using measures of population density, urbanization, and daily commuting [33]. Patients who live in rural and highly rural areas were combined and categorized as “rural.” Living in a rural area was assigned a value of 0 and living in an urban area was assigned a value of 1.

#### SM

Patients’ use of SM was quantified in 2017, the year prior to survey data collection, to enable us to evaluate the association between SM use (in 2017) and subsequent diabetes management (in late 2017/early 2018) and glycemic control (in 2018). We counted how many months of the year a patient sent at least one SM. SM use had a possible range of 0 to 12, where 0 reflected no months of SM use, and 12 reflected sending at least one secure message every month of the year.

To further understand patients’ use of SM, we coded the qualitative content of each SM in accordance with published coding methods [34], which have previously been used to code SM [24]. In addition, we coded each message using binary indicators for whether the messages were related to each of the following health topics: diabetes-related content, blood pressure, cholesterol, physical activity, diet/nutrition, and mental health. All messages were double-coded by 2 of 3 trained research team members who met regularly to discuss questions, reach agreement on any coding discrepancies, and refine the coding categories. Message codes were collapsed at the thread; that is, if a patient engaged in at least one message about diabetes, the entire message thread was coded as such. Patients were coded as having either engaged in at least 1 thread about a health topic or none. Additionally, as part of the larger study, we conducted qualitative interviews with 40 of the survey respondents [32]. We examined these interviews to further understand rural patient’s perceptions and use of SM.

#### Diabetes Self-management

Diabetes self-management behaviors were measured with the Diabetes Self-Management Questionnaire (DSMQ) [35]. The DSMQ is a global measure of diabetes self-management comprising 16 items to assess activities related to glycemic control in patients with diabetes (eg, “I strictly follow the dietary recommendations given by my doctor or diabetes specialist”; “I do regular physical activity to achieve optimal blood sugar levels”; and “I keep all of my doctors’ appointments recommended for my diabetes treatment”). The questionnaire asks participants to rate each item on a scale from 0 (does not apply to me) to 3 (applies to me very much). From the 16 items, a composite score was calculated as the average of 4 subscales, including glucose management, dietary control, physical activity, and health care use, and could range from 0 to 10. Higher values indicate greater engagement in self-management. The DSMQ has been shown to be significantly correlated with HbA_1c_ levels [35].

#### Glycemic Control

Glycemic control was defined as the estimated percentage of time in control (TIC) over the course of 2018 based on HbA_1c_ measurements (A_1c_%TIC). Patients’ HbA_1c_ measurements for 2018 were obtained from the CDW. We calculated A_1c_%TIC using the Rosendaal method [36], using linear interpolation to assign a value to each day between patient’s successive HbA_1c_ measurements. After interpolation, the percentage of 2018 during which the interpolated HbA_1c_ values within the region of control (ie, HbA_1c_<8.0%) were calculated.

#### Covariates

Covariates included age (measured in years), annual income in late 2017 or early 2018, and in-person health care utilization in 2017. Annual income was self-reported on a 16-category scale ranging from less than US $5000 to more than US $150,000. We dichotomized annual income using a median split of less than US $35,000 (46% of the sample) and US $35,000 or more (53.6%). The number of days a patient had a VA primary care visit in 2017 was used to measure in-person health care utilization.

### Analyses

**Figure 1:**
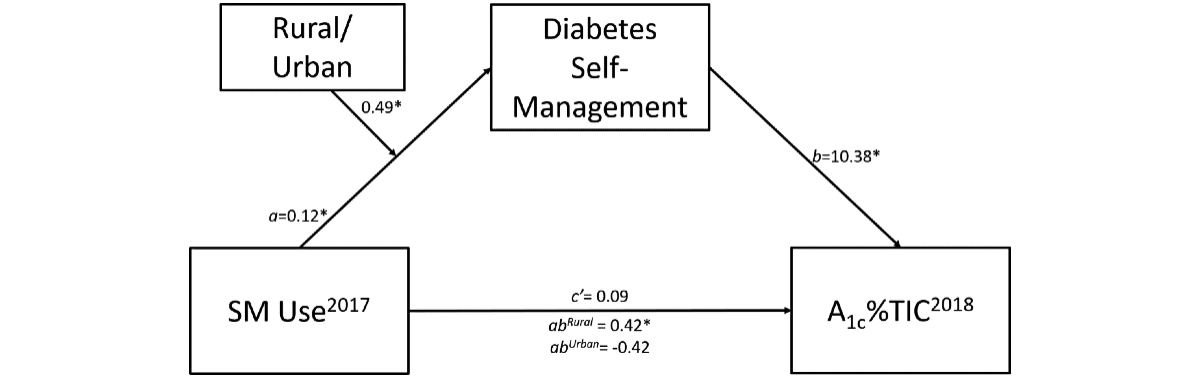
Moderated mediation between secure messaging (SM) use in 2017 and percent time in control of hemoglobin A_1c_ in 2018 (A_1c_%TIC), via diabetes self-management, moderated by rurality. Numbers represent parameter estimates. Model adjusts for age, gender, and income. *95% CI does not include 0.00 and *P*<.05.

We performed 2-tailed *t* tests, chi-square tests, and correlation analyses to examine differences between rural and urban participants and relationships between covariates and model measures. Moderated mediation was used to address the first 2 study objectives. Moderated mediation (Figure 1) estimates the indirect effect (SM use on A_1c_%TIC through diabetes self-management; research objective 1), and whether this indirect effect is conditional on values of a moderator (rurality; research objective 2). Analyses were conducted using Hayes’ PROCESS model in the SAS Enterprise Guide [37]. Moderation of the mediation model by rurality was assessed by calculating the index of moderated mediation [38] between rurality and the indirect effect between SM months in 2017 and A_1c_%TIC in 2018. The index of moderated mediation with a dichotomous moderator is defined as the difference in the indirect effects, or mediated effects, between the 2 levels of the moderator (rural and urban). The test of this index is assessed by generating a bootstrap 95% CI of the difference in indirect effects across moderator groups. Effects were considered significant if the 95% CI did not include 0.00 (*P*<.05). Qualitative analysis of the SM was used to further understand the nature of the secure message content and patient perceptions of SM (research objective 3).

## Results

We mailed 1200 surveys and received 448 (37%) responses. Table 2 describes the respondent sample in 2017, of whom just over half (51%) lived in rural areas. Most (94%) were male, and just over half (54%) reported an annual income above US $35,000. The mean age of survey respondents was 66.4 years (SD 7.5 years, range 34-88 years). In-person health care utilization ranged from 0 to 54 in-person visits; 52% (n=231) of the sample had 8 (median) or fewer in-person visits. As a population, they spent approximately half of their time in control in 2018 (mean A_1c_%TIC 52.6%, SD 43.6%, range 0%-100%). Their use of SM ranged from 0 to 12 months (mean 6.7 months, SD 3.1 months) in 2017. On average, patients reported relatively high levels of diabetes self-management (mean 7.9, SD 0.9, range 5.5-9.5). Rural and urban veterans were similar in income, age, A_1c_%TIC, in-person health care utilization, diabetes self-management score, and number of months using SM. SM use was significantly correlated with more in-person health care utilization (*r*=0.7, *P*<.001). Diabetes self-management was significantly correlated with a higher A_1c_%TIC in 2018 (*r*=0.21, *P*<.001).

**Table 2:** Respondent characteristics by rurality (N=446).

Characteristics	All	Rural (n=228)	Urban (n=218)	*P* value^a^
Male, n (%)	418 (94)	214 (94^b^)	204 (94^c^)	0.90
Income <US $35,000, n (%)^d^	207 (46)	111 (49^b^)	96 (44^c^)	0.33
Age, mean (SD)	66.4 (7.5)	66.3 (7.3)	66.5 (7.7)	0.82
2017 In-person primary care visits, mean (SD)	10.1 (7.8)	9.5 (7.0)	10.6 (9.5)	0.12

^a^Rural vs urban respondents.

^b^Percentage values are based on a total value of 228 respondents.

^c^Percentage values are based on a total value of 218 respondents.

^d^Income from the survey was for late 2017 or early 2018 based on when respondents completed their survey.

### Diabetes Self-management

More months using SM was significantly and positively associated with greater diabetes self-management (*B*=0.12, 95% CI 0.033-0.212; *P*=.007; *a* in Figure 1). Rurality influenced the strength of the relationship between SM use and diabetes self-management (*B*=–0.08, 95% CI –0.138 to –0.026; *P*=.005). When we examined the conditional effects of SM on diabetes self-management for rurality, there was a trend to a significant positive relationship between SM and diabetes self-management for rural patients (*B*=0.04, 95% CI –0.001 to 0.083; *P*=.06) and a trend toward a negative relationship between SM and diabetes self-management among urban patients (*B*=–0.04, 95% CI –0.080 to 0.002; *P*=.06).

### Glycemic Control

Patients who reported greater diabetes self-management had significantly higher A_1c_%TIC (ie, more time in control of their diabetes throughout the year; *B*=10.38, 95% CI 5.539-15.217; *P*<.001; *b* in Figure 1). There was no direct effect of SM use on A_1c_%TIC (*B*=0.09, 95% CI –1.463 to 1.651; *P*=.91; *c’* in Figure 1). However, there was a conditional indirect effect between SM use and A_1c_%TIC, via diabetes self-management for rural patients (*B*=0.42, 95% CI 0.004-0.927; Table 3 and *ab*^Rural^ in Figure 1). This conditional indirect effect represents the change in A_1c_%TIC for every month of SM use, mediated by diabetes self-management. Among urban patients, there was no indirect effect between SM use and A_1c_%TIC via self-management (*B*=–0.42, 95% CI –1.039 to 0.056; *ab*^Urban^ in Figure 1). The index of moderated mediation (ie, the difference between rural and urban indirect effects) was significant (index=–0.85, 95% CI –1.64 to –0.23).

**Table 3:** Moderated mediation analyses.

		*B* (95% CI)	*P* value
**Model to predict diabetes self-management**			
	Constant	5.81 (4.82 to 6.81)	<.001
	Secure messaging use during 2017	0.12 (0.03 to 0.21)	.007
	Rurality	0.49 (0.08 to 0.90)	.02
	Secure messaging use during 2017*Rurality	–0.08 (–0.13 to –0.03)	.005
	Secure messaging use during 2017*Rural	0.04 (0.00 to 0.08)	.06
	Secure messaging use during 2017*Urban	–0.04 (–0.08 to 0.00)	.06
	Age	0.02 (0.01 to 0.03)	.003
	In-person primary care visits in 2017	0.01 (–0.01 to 0.02)	.29
	Income (reference=<US $35,000)	0.16 (–0.01 to 0.34)	.08
**Model to predict the percent time in control of hemoglobin A_1c_ in 2018**			
	Constant	–19.13 (–69.74 to 31.49)	.46
	Direct effect of secure messaging use during 2017 on the percent time in control of hemoglobin A_1c_ in 2018	0.09 (–1.46 to 1.65)	.91
	Diabetes self-management	10.38 (5.54 to 15.22)	<.001
	Age	–0.17 (–0.75 to 0.41)	.56
	In-person primary care visits in 2017	0.11 (–10.70 to 6.72)	.65
	Income (reference=<US $35,000)	–1.99 (–10.70 to 6.72)	.65
**Indirect effects of Rurality on the percent time in control of hemoglobin A_1c_ in 2018**			
	Rural	0.42 (0.01 to 0.92)	—^a^
	Urban	–0.42 (–1.03 to 0.05)	—

^a^—: not determined.

### Sensitivity Analysis

This study modeled SM use in 2017 and A_1c_%TIC in 2018. Had we examined both SM use and glycemic control in the same year, we would have risked potentially having some participants with SM data toward the end of the year and HbA_1c_ measurements in the beginning of the year. These data would not be consistent with the hypothesized temporal nature of the analysis. However, as sensitivity analysis, we compared SM use in 2017 and 2018. SM use in 2017 and 2018 were significantly correlated (*r*=0.53, *P*<.001). Additionally, we ran the moderated mediation model using both SM use and A_1c_%TIC in 2018. A similar pattern of results occurred in a moderated mediation analysis that examined both SM use and A_1c_%TIC simultaneously in 2018. Further information is included in Multimedia Appendix 1.

### SM Content

Qualitative analysis of the SM content revealed that significantly more rural participants (77%, n=177) discussed diabetes-related content in at least one SM thread than urban participants (67%, n=146; *P*=.01). There were no other significant differences between the proportion of urban and rural participants who engaged in at least one thread related to other health topics codes. Semistructured interviews with a subset of survey respondents further expanded on how rural patients perceived SM and were using SM (Table 4). Patients consistently expressed how SM helped them communicate with their clinical teams. Rural patients indicated that SM was a convenient tool to support tasks pertinent to effective diabetes self-management. For example, one patient reported that SM was a more reliable form of communication than through a cell phone to set up appointments or medication renewal requests. Patients also indicated they were able to use SM to communicate their diabetes-related equipment needs with their clinical team*.* Patients also reported that SM allowed them to communicate with various members of their clinical team.

**Table 4:** Qualitative themes and representative quotes.

Communication theme	Quote
Reliability	*Cellphones don’t work real [sic] well around here, you have to be in certain areas. There’s lots of dead spots, like hundreds of miles of it…It works better for [hospital] to use secure messaging to set up appointments. I’ve used them to talk to [the clinic], I’ve used them to talk to my provider a couple of times when I needed prescriptions changed or stuff like that.* [Rural male, 69 years]
Communicate needs rapidly	*I’ve got central tremors and…I’m shaking and I can’t get the syringe in the bottle you know. So I just sen[t]… a SM. I said Dr. [name] I want to get pens again I can’t do it. Within 48 hours, I’m serious, literally. I had pens delivered to my front door you know. Just absolutely wonderful as far as I’m concerned.* [Rural male, 54 years]
Facilitates communication with team	*Once I tried to message them or if I sent a message to the nursing staff then the next time I got on there, there was a connection for me to, you know, to send a message directly to my pharmacist...* [Rural female, 65 years]

## Discussion

### Principal Findings

In a population of veterans with diabetes, we examined the relationship between the use of SM and percent time in glycemic control, whether diabetes self-management behaviors mediated this relationship, and if the use of SM is beneficial for those both living in urban and rural areas. This study leveraged mixed methods to quantify these relationships through a moderated mediation analysis, examine how patients with diabetes use SM through a message content analysis, and learn from patients through qualitative interviews. Moderated mediation analysis revealed that the relationship between the use of SM, diabetes self-management, and A_1c_%TIC was influenced by rurality. Among rural patients, increased use of SM was associated with a higher A_1c_%TIC through diabetes self-management. The mediation of SM and A_1c_%TIC through diabetes self-management was not found among urban patients. This finding does not indicate that SM is not necessarily beneficial for urban patients; rather, it indicates that SM may help support rural patients’ diabetes self-management efforts to a greater extent than among urban patients. In addition to the challenges of effective diabetes management, rural patients face additional barriers including limited access to diabetes education and clinical services, limited cell phone coverage and internet access, limited transportation, and long travel distances [39]. It is possible that the enhanced clinical access afforded by SM may not influence self-management among urban patients who do not face the same access barriers as their rural counterparts [10]. SM offers rural patients means to overcome many of these barriers.

Our quantitative analysis included all SM communication (ie, not just diabetes-specific SM) as many different subjects, such as messages about hypertension or physical activity, are likely to be helpful for diabetes management. We used qualitative analyses to further explore the ways in which rural patients leverage SM for diabetes self-management. Rural patients were more likely than their urban counterparts to communicate via SM with their health care team about diabetes-related content, which may be associated with more effective diabetes management efforts. While messages about other health topics may be just as important for diabetes management, there were no significant differences in the frequency in which these other health topics were discussed between rural and urban patients. Additionally, participant interviews revealed insights into some of the benefits SM affords rural participants, such as SM being a more reliable and convenient means to communicate with various members of their clinical team to engage in activities important for diabetes management (eg, appointment requests, medication renewals, and equipment requests).

This relationship between increased health care team access and greater self-management aligns with previous research; a systematic review evaluating technology-enabled diabetes self-management support concluded that 2-way communication between the patient and clinical team was an essential component for improved HbA_1c_ [40]. Patients who use web-based portals and SM can communicate with their team more regularly, as needed, and potentially reduce the need for in-person visits. Reports on the relationship between SM and in-person health care utilization are inconsistent. For example, we found that greater use of SM was positively associated with more in-person health care utilization, whereas other recent work has found that use of SM was associated with a decrease in in-person utilization [41]. It is difficult to disentangle if patients are using SM in place of in-person care, or if they are using SM because of an upcoming or recent in-person visit (eg, following up on a new medication). Owing to this potential confounder, we included in-person primary care visits as a covariate in our model to control for health care utilization and possible confounding by indication.

### Implications

More consistent use of SM, particularly SM related to diabetes, can help overcome commonly reported regional disparities in diabetes self-management and glycemic control. Despite the benefits of SM for diabetes self-management and glycemic control in rural veterans with diabetes, rural patients are less likely to manage personal health information on the internet or communicate through the internet with their providers [30]. External support from a patient’s clinical team has been identified as a key facilitator of diabetes self-management [39], though such support is less available for patients with limited access to in-person visits. Fortunately, virtual modalities such as web-based patient portals and features including SM can provide easily accessible support for effective diabetes self-management. It is critical to identify methods that will promote patients’ use of web-based portals for better chronic disease management. Technology-based approaches and interventions are widely accepted for promoting diabetes self-management in rural communities [42]. Additionally, we previously found that as little as one team-initiated secure message was significantly associated with better diabetes self-management [25]. Providers may find that encouraging patients, particularly rural patients, to use SM may significantly improve their diabetes self-management and outcomes.

SM has the potential to reach an ever-increasing number of patients. As of July 2021, 3.7 million veterans (more than half of active VA patients) were registered portal users, of which 1.4 million were active users of SM. Increasing SM use can be considered a high-reach, light-touch intervention with the potential to improve population health. Understanding the benefits of modalities that can provide more accessible diabetes self-management support not only has implications for rural patients who typically face barriers accessing in-person health care owing to long travel distances—these findings also support the value of encouraging SM use when in-person visits are not feasible. During the COVID-19 pandemic, VA facilities were directed to convert in-person to virtual care whenever clinically appropriate [43] and for rural patients in particular [44]. Use of SM can help maintain patient-provider communication and support disease self-management when patients cannot access in-person care. Emerging evidence suggests that disparities in rural patients’ access to telemedicine, including video visits and portals, have persisted despite dramatic increases in adoption [45]. Our findings suggest that efforts to reduce these disparities are important not only to improve equity but also to support improved outcomes.

### Limitations and Future Directions

This study has some limitations. For one, this sample purposively surveyed patients who were both recent and repeated users of patient portals; it does not speak to the potential benefit of SM in those who have never used portals. Those who responded to our study may, as a group, have had better self-management than the average patient with diabetes. Indeed, our sample scored higher on the DSMQ than other populations, though not outside the SD [35]. Similarly, as is common in many US Veteran studies, our sample size was mostly male, which limits the potential generalizability of these findings to females and non-Veterans.

The current analysis examined self-management as a composite score. Future research may examine the relationship between SM use and various self-management behaviors, and if certain self-management behaviors are more important in the relationship between SM use and A_1c_%TIC. Finally, another limitation is the cross-sectional and observational nature of the study. Our mediation model allows us to begin to think about the causal nature of these relationships. Future studies might benefit from interventional designs that examine changes to diabetes self-management and glycemic control after initiating SM use compared to a sample who have never used SM.

### Conclusions

On average, patients with diabetes who live in rural areas are disproportionately affected by diabetes, in part owing to their limited access to health care. Among rural patients, greater use of SM was associated with better diabetes self-management, which was associated with better glycemic control. This was not observed among urban patients. Rural patients with diabetes may benefit significantly from using SM to support their diabetes self-management and diabetes-related outcomes. Encouraging patients to ask questions between visits, or reaching out to them directly via SM, are examples of light-touch interventions with potential to improve outcomes for millions of patients with diabetes who lack ready access to in-person care.

## Appendix

Multimedia Appendix 1 Supplementary sensitivity analysis.

## Abbreviations

A_1c_%TIC: percentage of time in control over the course of the year based on hemoglobin A_1c_ measurements
CDW: Corporate Data Warehouse
DSMQ: Diabetes Self-Management Questionnaire
HbA_1c_: hemoglobin A_1c_
MHV: My Health*e*Vet
RUCA: Rural-Urban Commuting Areas
SM: secure messaging
TIC: time in control
VA: Veterans Affairs
VHA: Veterans Health Administration
