# Supplementary material for: Differences in Secure Messaging, Self-management, and Glycemic Control Between Rural and Urban Patients: Secondary Data Analysis
Source: JMIR Diabetes. 2021 Nov 19;6(4):e32320. doi: 10.2196/32320 (PMC8663667; doi:10.2196/32320)
Supplement: Multimedia Appendix 1 [file diabetes_v6i4e32320_app1.docx]

Differences in Secure Messaging, Self-management, and Glycemic Control between Rural and Urban Patients: Secondary Data Analysis

Supplementary Materials

### Sensitivity Analysis

As a sensitivity analysis, we ran a moderated mediation analysis of SM use in 2018 and A1c%TIC in 2018, with diabetes self-management as a mediator and rurality as a moderator. This model used income and race (collected via survey at the end of 2017 and beginning of 2018), and in-person healthcare utilization from 2018 as a covariate.

### Diabetes Self-Management

More months using SM in 2018 was significantly and positively associated with greater diabetes self-management (*B* = 0.10, 95% CI [0.018,0.184], *P* = .017). Rurality influenced the strength of the relationship between SM use and diabetes self-management (*B* = -0.06, 95% CI [-0.118, -0.010], *P* = .020). When we examined the conditional effects of SM on diabetes self-management for rurality, there was a trend to a significant positive relationship between SM and diabetes self-management for rural patients (*B* = 0.04, 95% CI [-0.001, 0.075], *P* = .057). There was no significant relationship between SM and diabetes self-management among urban patients (B = -0.03, 95% CI [-0.07, 0.015], *P* = .212).

### Glycemic Control

Patients who reported greater diabetes self-management had significantly higher A_1c_%TIC (i.e., more time in control of their diabetes throughout the year; *B* = 10.52, 95% CI [5.683, 15.352], *P* < .001). There was no direct effect of SM use on A_1c_%TIC (*B* = -0.34, 95% CI [-1.787, 1.106], *P* = .644), nor was evidence of a conditional indirect effect between SM use and A_1c_%TIC, via diabetes self-management for rural patients. The index of moderated mediation (i.e., the difference between rural and urban indirect effects) was significant (Index = -0.67, 95% CI [-1.414, -0.090]).

| Table 1s. Supplementary moderated mediation analyses between SM Use in 2018 and A1c%TIC in 2018. | | | | |
| --- | --- | --- | --- | --- |
|  |  | 95% CI | |  |
|  | B | Lower | Upper | *P* |
|  |  |  |  |  |
| Model to Predict Diabetes Self-management |  |  |  |  |
| Constant | 5.97 | 4.98 | 6.96 | <.001 |
| SM Use^2018^ | 0.10 | 0.02 | 0.18 | 0.017 |
| Rurality | 0.38 | -0.01 | 0.76 | 0.055 |
| SM Use^2018^ * Rurality | -0.06 | -0.12 | -0.01 | 0.020 |
| SM Use^2018^ * Rural | 0.04 | 0.00 | 0.08 | 0.057 |
| SM Use^2018^ * Urban | -0.03 | -0.07 | 0.02 | 0.212 |
| Age | 0.02 | 0.01 | 0.03 | 0.003 |
| In-person Primary Care Visits^2018^ | 0.00 | -0.00 | 0.01 | 0.696 |
| Income (ref=<$35,000) | 0.16 | -0.02 | 0.34 | 0.082 |
| Model to Predict A_1C_%TIC^2018^ |  | | | |
| Constant | -14.04 | -64.81 | 36.73 | 0.587 |
| SM Use^2018c'^ | -0.34 | -1.79 | 1.11 | 0.644 |
| Diabetes Self-management | 10.52 | 5.68 | 15.35 | <.001 |
| Age | -0.18 | -0.76 | 0.40 | 0.536 |
| In-person Primary Care Visits^2018^ | -0.10 | -0.66 | 0.465 | 0.728 |
| Income (ref=<$35,000) | -2.70 | -11.40 | 6.00 | 0.542 |
| Indirect effects of Rurality on A_1C_%TIC^2018^ |  |  |  |  |
| Rural | 0.39 | -0.00 | 0.89 |  |
| Urban | -0.28 | -0.82 | 0.21 |  |
| Notes: SM Use^2018^ = Secure Messaging Use in Months during 2018; A_1C_%TIC^2018^ = Percent Time in Control of HbA_1c_ in 2018; ^c'^Direct Effect of SM Use^2018^ on A_1C_%TIC^­­^. | | | | |
